# Opinions of nephrologists on the efficacy and tolerance of statins in hemodialysis patients

**DOI:** 10.1080/0886022X.2016.1260032

**Published:** 2016-11-25

**Authors:** Ewa Budzisz, Michał Nowicki

**Affiliations:** Department of Nephrology, Hypertension and Kidney Transplantation, Medical University of Lodz, Lodz, Poland

**Keywords:** Hemodialysis therapy, statins, drug safety, survey, expert opinion

## Abstract

Large randomized controlled trials have not confirmed the effects of statin therapy on reduction of cardiovascular morbidity and mortality in end-stage kidney disease, despite that statins are still widely prescribed by nephrologists to chronic dialysis patients. The aim of the study was to analyze the attitudes of nephrologists towards statin use in hemodialysis patients. Self-designed questionnaire, containing 18 questions, was distributed among 115 nephrologists. The survey contained description of the results of 3 largest statin trials in nephrology. The questions referred to the interpretation of trial results and the safety and efficacy of statin therapy and dose adjustments required in dialysis patients. 83% among 72 nephrologists who returned the questionnaire prescribed statins to their dialysis patients for secondary prevention of cardiovascular events. 90% prescribed atorvastatin. 64% nephrologists did not modify statin dose at the start of hemodialysis treatment and 47% before elective surgery. Liver disease was indicated as a main reason for dose modification in hemodialysis patients. Statin-induced myopathy was observed by 65% nephrologists and 61% reported a case of increased liver enzymes. 51% of nephrologists did not routinely discuss the possible benefits and risks of statin therapy with their patients. Statins are still widely prescribed and considered safe and effective lipid-lowering therapy in dialysis patients by most nephrologists.

## Introduction

Cardiovascular mortality is exceedingly high in chronic hemodialysis patients (HD).^1^ Nearly all dialysis patients have multiple comorbidities including arterial hypertension, coronary artery disease, diabetes and cerebrovascular disease.^2^ The increased risk of atherosclerotic cardiovascular disease in the patients with chronic kidney disease (CKD) poorly correlates with increased serum lipid concentration indicating a complex pathogenesis of vascular pathology in this population.^3^ The recent analysis provided the evidence that statins retain their beneficial effect on cardiovascular morbidity and mortality in patients with CKD not receiving dialysis.^4^ Therefore, the Kidney Disease Improving Global Outcomes (KDIGO) Lipid Work Group has recommended statins for all non-dialysis adults with CKD older than 50 years. KDIGO guideline recommends a routine assessment of mortality risk of patients with CKD.^5^

In contrast to the general population^6^ and non-dialysis patients with chronic kidney disease,^4^ the results of large randomized clinical trials^7–9^ have not confirmed significant effects of statin therapy on the reduction of cardiovascular morbidity and mortality in chronic dialysis patients.^10^ Therefore, KDIGO experts do not recommend a routine initiation of statin therapy in this population.^11^ However, the clinicians should use their clinical judgment and weigh the risks and benefits when deciding whether to initiate or continue statins in chronic hemodialysis patients. Statin may be warranted for secondary prevention of cardiovascular events or in hemodialysis patients who have a longer life expectancy^12^ and in those awaiting kidney transplantation.^13^

Interestingly, despite the lack of apparent benefit statins are still prescribed for many dialysis patients.^14^ The aim of the study was to analyze the attitudes of board-certified nephrologists towards statin use in chronic hemodialysis patients.

## Material and methods

A self-designed, 18-item questionnaire (Table 1) was distributed among 115 nephrologists from 14 dialysis center in Central Poland, who were directly involved in the care of chronic dialysis patients. The mean time of practicing nephrology by the nephrologists was 15 ± 9 years.

The questionnaire included a short description of the goals and an invitation to share the opinions about the treatment with statin. The introduction was followed by the short description of the results of three largest randomized, prospective, multicenter studies that included chronic dialysis patients (4D—Deutsche Diabetes Dialyze Studie, AURORA—study to evaluate the Use of Rosuvastatin in subjects On Regular hemodialysis: an Assessment of survival and cardiovascular events; and SHARP-Study of Heart and Renal Protection).^7–9^ The information about the RCTs provided to the nephrologists (Table 2) included a number of subjects who received the therapy in each trial, size of a control group, study duration, primary outcomes with relative risk, the 95% confidence interval and significance.

**Table 1.** The list of the questions included in the survey.

Number of question	
	**Professional experience in nephrology.**
1	How long have you practiced nephrology?
2	How many hemodialysis patients are cared by you?
3	How many peritoneal dialysis patients are cared by you?
4	What percent of dialysis patient carrying by you receive lipid lowering therapy?
	**Lipid lowering therapy in dialysis patients according to RCTs and professional experience.**
5	Which groups of lipid lowering drugs do you use in treatment of dialysis patients?
6	Is it still advisable to use statins in dialysis patients providing results of RCTs?
7	Why statins regimen could be important for dialysis patients? Pleas, justify your answer.
	**Blood tests recommended to initiate and monitor statin therapy**
8	Which laboratory tests do you prescribe before beginning of statins therapy?
9	Which laboratory tests and how often do you prescribe to control efficacy and safety of statins regimen?
	**Statin therapy intended for hemodialysis patient**
10	Which statins do you use most frequently in dialysis patients?
11	Which dose of statin is safe and efficient in treatment of dialysis patient?
12	Do you use different dosage of statin in early stages of Chronic Kidney Disease in comparison to dialysis patients?
	**Side effects**
13	Which side effects of statins have you seen in dialysis patients?
	**Adjustment of the statin dose**
14	Which lipid lowering therapy would you choose in case of co-morbidity with Diabetes Mellitus in dialysis patients?
15	Which lipid lowering therapy would you choose before extensive surgery in dialysis patients?
16	Would you change statins regimen before surgical creation of arteriovenous fistula?
17	Which lipid lowering therapy would you choose in case of co-morbidity with chronic virusal hepatitis?
	**Personal attitude of the nephrologist to inform their patients about the effect of statin therapy**
18	What is your attitude to inform dialysis patients about efficacy and safety of statins before beginning of lipid lowering therapy?

**Table 2.** Summary of the design and results of large randomized controlled trials, i.e., 4D, AURORA and SHARP included in the introductory part of the questionnaire.

Name of the study	Investigational drug	Primary end-point	Number of events/number of patients	Control group	Relative risk/ hazard ratio (95%Cl)	*p* Values	Studied patients
4D	Atorvastatin	CV death, nonfatal MI, stroke	226/619	243/636	RR: 0.92 (0.77–1.10)	.37	Dialysis patients with type 2 diabetes
AURORA	Rosuvastatin	CV death, nonfatal MI, stroke	396/1391	408/1385	HR: 0.96 (0.84–1.11)	.59	Dialysis patients
SHARP	simvastatin & ezetimibe	CV death, MI, non-haemorrhagic stroke, or any revascularisation	526/4650	619/4620	RR: 0.83 (0.74–0.94)	0.0021 (significant only in non-dialysis patients)	Dialysis and non-dialysis patients

The questionnaire was divided into four different sections. The first group of questions referred to the general medical experience. The doctors were asked of a time of practicing nephrology, a number of patients treated with hemodialysis that are under their direct medical care and a percentage of the patients prescribed the lipid-lowering therapy. The second section contained the questions focused on the attitude towards statin therapy with respect to the results of large RCTs. The next group of questions referred to the laboratory tests that are routinely done before initiation and for the monitoring of the statin therapy. The doctors were also asked about the International Nonproprietary Name (INN) of the statin they prescribe most often to dialysis patients and dose range that they consider as safe and effective. The next question referred to the side effects related to statin use that were observed by the nephrologists. That was followed by the questions about the attitude towards the modification of statin therapy in patients with diabetes mellitus or viral hepatitis. In this part, the nephrologists were also asked about a need to change the dose of statin in dialysis patients before an extensive surgery or local surgery to create an arteriovenous fistula. The final question referred to a practice to inform dialysis patients about the safety and efficacy of statins therapy before the start of lipid-lowering therapy.

The standard methods of descriptive statistics were used. The results were presented as mean ± SD or as frequency (%).

## Results

One hundred fifteen nephrologists were asked to take part in the survey. Seventy-two (63%) provided the responses. The doctors were approached in different ways. Forty questionnaires were directly delivered to dialysis centers by a member of the study team. Twenty-nine of those questionnaires were completed and handed back to researchers. Sixty questionnaires were sent to dialysis centers by regular mail following the initial invitation by phone. Thirty-five of them were filled and sent back to researchers. Fifteen questionnaires were distributed during the medical conference organized on the occasion of the “World Kidney Day”. Only four questionnaires of those questionnaires were completed and handed back to researchers at the end of the conference. Another four questionnaires were sent back after the meeting.

The mean time of practicing nephrology after receiving the certification was 15 ± 9 years with the range of practice from 1 to 30 years. The mean number of chronic hemodialysis patients under a direct care of the nephrologist was 46 ± 34. Thirty-three percent of the nephrologists took also care of peritoneal dialysis patients.

Having considered the results of seminal clinical trials and professional experience 83% of nephrologists expressed the opinion that they would still use statins for the treatment of hemodialysis patients. Twenty-six percent of nephrologists expressed the opinion that statins may be particularly beneficial in hemodialysis patients who have a history of myocardial infarction (MI) (15%), stroke (6%), transient ischemic attack (TIA) (4%) or coronary intervention (3%), and in patients with ischemic heart disease (7%), diabetes (3%) or atherogenic dyslipidemia (3%). Lipid-lowering therapy, initiated before hemodialysis was listed as a main argument for continued use of statins during dialysis therapy (7%). Thirteen percent of nephrologists prescribed statins because they believed that they have not only lipid-lowering but also anti-inflammatory properties (4%), anticoagulant (1%) and antioxidant activity (3%), and may stabilize the atherosclerotic plaque (3%). Eleven percent of nephrologists answered that they prescribe statins to their dialysis patients because they are at a higher risk of atherosclerosis and cardiovascular events compared to the general population. Only 11% of the responders did not agree with the statement that statins lower the risk of cardiovascular events when used for primary prevention in dialysis patients. Seventeen percent of the doctors preferred not to use statins in hemodialysis patients having considered a lack of their apparent effect on cardiovascular endpoints. Despite that, 100% of the nephrologists who took part in the survey answered that they prescribe statins for hemodialysis patients and 43% would prescribe them with fibrates when needed.

All nephrologists control the general health status of the patients before the initiation of statin therapy. Most of the doctors assessed serum lipid profile (97%), liver enzymes (glutamic-pyruvic transaminase (GPT) activity (83%), glutamic-oxaloacetic transaminase (GOT) activity (72%) and creatine kinase (CK) activity (53%). Thirty-six percent of the doctors considered as appropriate to closely monitor serum potassium concentration before starting lipid-lowering therapy. Sixty percent monitored the safety of the therapy by checking GPT and GOT at least every 3 months. Lipid profile was assessed to monitor the effects of the therapy every 6 months by 1/3 of nephrologists. CK was measured to monitor side effects by 33% of nephrologists only if required.

Atorvastatin was chosen by 90% of nephrologists as an appropriate lipid lowering agent for hemodialysis patients mostly due to its small elimination with the kidneys. Forty-three percent of nephrologists also prescribed simvastatin. Sixty-seven percent of nephrologists supported the opinion that the dose of statin needs to be reduced in HD patients at least by about 25%. Sixty-three percent of nephrologists used the same dose of statin in all CKD stages and 25% reduced a dose of statin only in chronic dialysis patients.

The nephrologists were asked if they adjust a dose of statin in particular clinical situations. In their opinion, chronic hepatitis was a main reason for a reduction of the dose of statin. The other reasons included diabetes (13%), preparation to an extensive surgery (6%), preparation to arteriovenous fistula creation (4%). Statin therapy was adjusted to current levels of serum lipids in HD patients and cardiologist recommendation after a recent cardiac event (11%). Thirty-three percent of nephrologists modified statins in diabetic patients, part of them by reducing their dose but 7% increased the dose. Thirteen percent of nephrologists modified dietary recommendation, considered additional fibrate therapy or adjusted the dose of statin according to the rate of drug tolerance and efficacy. Chronic hepatitis was the main reason for the discontinuation of statin (30%) while 35% lowered the dose or adjusted the dose (20%) according to the activity of GPT and GOT or individual tolerance. An extensive surgery was another clinical situation that influenced statin prescription. Seventeen percent of nephrologists answered that the preparation to a major surgery was a reason to discontinue the therapy. Six percent decided only to lower a dose in such case. Seventy-eight percent of nephrologists did not modify the therapy and 30% ordered an additional laboratory work–up including serum GPT, GOT and potassium before dose modification.

**Figure 1.**
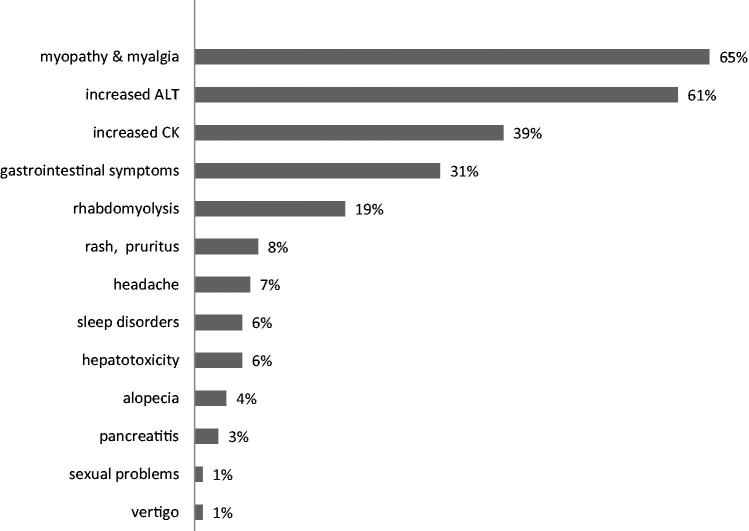
Adverse effects of statins reported by the nephrologists in their chronic dialysis patients.

The therapy with statin was associated with various adverse effects. Myopathy with myalgia and increased GPT were the most frequent side effects of statins that nephrologists were aware of. Adverse reactions of statins reported by the nephrologists are presented in Figure 1.

The final part of the questionnaire addressed the problem of the education and awareness of dialysis patients about efficacy and tolerance of statins. Most nephrologists (71%) informed their hemodialysis patients about an increased risk of cardiovascular events that have been related to their disease and condition including a risk of myocardial infarction or stroke. Almost half of the nephrologists informed the patients about the uncertain benefits of statins in hemodialysis population. According to 61% of nephrologists, it would be reasonable to inform the dialysis patients that they are particularly prone to the development of adverse effects of statins.

## Discussion

Previous studies and recent meta-analysis suggested that statins may be cost-effective for primary prevention in all patients with CKD excluding those on chronic dialysis.^15^ Statins are routinely used by most nephrologists in chronic dialysis patients despite the lack of an apparent benefit shown in the large randomized clinical trials.^11–14^ That prompted us to study the attitude of board-certified nephrologists to statin therapy. We tried to assume if there is a difference in prescribing statins by nephrologists for the primary or secondary prevention in chronic hemodialysis patients. We have designed a questionnaire that targeted doctors who are directly involved in the care of dialysis patients. We used a paper questionnaire which is a cheap way of acquiring data easy and comfortable for participant who can express their opinion without pressure of time.

We have chosen to survey board-certified nephrologists to get their opinion about statin therapy in dialysis patients. Doctors are the particular group of subject in medical research because of their low response rate in studies that are based on questionnaires.^16–18^ This fact makes the data collection difficult and requires a special effort from researchers to receive credible answers. We reached with our questionnaire about 25% of Polish nephrologists who treat dialysis patients what is probably a limitation of our study. We achieved a relatively high response rate (63%) when compared to other surveys carried out among physicians. In comparison a survey performed in Australia that also used a traditional paper questionnaire response rate was only 19.7%^18^ and in The Canadian National Physician Survey conducted among medical specialists, surgical specialists, family physicians, laboratory medicine specialists and internal medicine specialists where only 16% responded.^17^ The last study included also the nephrologists and a response rate among them was only 13%.^17^

The relatively high response rate in our study was probably due to the fact that the survey was brief. Our questionnaire contained only 18 questions. Survey length matters for response rates as it was in case of above mentioned study.^18^ The length of their questionnaire ranged from 58 questions in an eight-page booklet (for specialists-in-training) to 87 questions in a 13-page booklet (for specialists). The above-mentioned study performed among the Canadian physicians used a questionnaire which contained 24 questions and they accomplished low response rate among nephrologists.^17^

The idea of investigating opinion of medical professionals is not popular. We found only 3 surveys based on similar idea among which only one referred to the treatment with statins.^17–19^ Lack of validation of our questionnaire is a limitation of the study. The group included the nephrologists with different clinical experience but a size of the population did not allow us to perform a subgroup analysis. Particularly interesting would be to study whether the responses were dependent on the size of a center or its location and distance from an academic center. The results can differ between rural areas and major cities as it has been shown in previous surveys performed among healthcare specialists.^18–20^

The major finding of our survey is that statins are still considered safe and effective lipid-lowering therapy in dialysis patients by most board-certified nephrologists despite the lack of convincing evidence from hard endpoint-oriented trials.
